# Rural–urban disparities in the incidence and treatment intensity of periodontal disease among patients with diabetes

**DOI:** 10.3389/fpubh.2023.1241150

**Published:** 2023-09-06

**Authors:** Hsueh-Fen Chen, Huey-Er Lee, I-Te Chen, Yu-Ting Huang, Pei-Shan Ho, Saleema A. Karim

**Affiliations:** ^1^Department of Healthcare Administration and Medical Informatics, College of Health Sciences, Kaohsiung Medical University, Kaohsiung, Taiwan; ^2^Division of Medical Statistics and Bioinformatics, Department of Medical Research, Kaohsiung Medical University, Kaohsiung, Taiwan; ^3^Center for Big Data Research, Kaohsiung Medical University, Kaohsiung, Taiwan; ^4^Department of Dentistry, Yuan's General Hospital, Kaohsiung, Taiwan; ^5^Department of Oral Hygiene, College of Dental Medicine, Kaohsiung Medical University, Kaohsiung, Taiwan; ^6^School of Dentistry, College of Dental Medicine, Kaohsiung Medical University, Kaohsiung, Taiwan; ^7^Department of Health Administration, College of Health Professions, Virginia Commonwealth University, Richmomd, VA, United States

**Keywords:** type 2 diabetes, periodontal disease, rural–urban disparities, unmet dental needs, underutilization

## Abstract

**Background:**

Diabetes threatens population health, especially in rural areas. Diabetes and periodontal diseases have a bidirectional relationship. A persistence of rural–urban disparities in diabetes may indicate a rural–urban difference in periodontal disease among patients with diabetes; however, the evidence is lacking. This retrospective study aimed to investigate rural–urban discrepancies in the incidence and treatment intensity of periodontal disease among patients who were newly diagnosed with type 2 diabetes in the year 2010.

**Methods:**

The present study was a retrospective cohort design, with two study samples: patients with type 2 diabetes and those who were further diagnosed with periodontal disease. The data sources included the 2010 Diabetes Mellitus Health Database at the patient level, the National Geographic Information Standardization Platform and the Department of Statistics, Ministry of Health and Welfare in Taiwan at the township level. Two dependent variables were a time-to-event outcome for periodontal disease among patients with type 2 diabetes and the treatment intensity measured for patients who were further diagnosed with periodontal disease. The key independent variables are two dummy variables, representing rural and suburban areas, with urban areas as the reference group. The Cox and Poisson regression models were applied for analyses.

**Results:**

Of 68,365 qualified patients, 49% of them had periodontal disease within 10 years after patients were diagnosed with diabetes. Compared to urban patients with diabetes, rural (HR = 0.83, 95% CI: 0.75–0.91) and suburban patients (HR = 0.86, 95% CI: 0.83–0.89) had a lower incidence of periodontal disease. Among 33,612 patients with periodontal disease, rural patients received less treatment intensity of dental care (Rural: RR = 0.87, 95% CI: 0.83, 0.92; suburban: RR = 0.93, 95% CI: 0.92, 0.95) than urban patients.

**Conclusion:**

Given the underutilization of dental care among rural patients with diabetes, a low incidence of periodontal disease indicates potentially undiagnosed periodontal disease, and low treatment intensity signals potentially unmet dental needs. Our findings provide a potential explanation for the persistence of rural–urban disparities in poor diabetes outcomes. Policy interventions to enhance the likelihood of identifying periodontal disease at the early stage for proper treatment would ease the burden of diabetes care and narrow rural–urban discrepancies in diabetes outcomes.

## Introduction

Diabetes is a growing financial and medical challenge to public health worldwide. In 2021, the cost of diabetes care was USD 699 billion globally (1). Diabetes contributes to several life-threatening conditions, including cardiovascular diseases (e.g., stroke and heart attacks) in the macrovascular system and several pathological changes in the microvascular system, which leads to disability, such as lower-extremity amputation due to neuropathy, blindness due to retinopathy, and renal failure due to nephropathy (2). For example, about 18.1 million individuals suffered disabilities due to diabetes-related lower-extremity complications globally in 2016 (3). Also, in 2019, about 1.5 million deaths were directly related to diabetes, and 48% died before 70 years old (4). Although these complications and premature deaths can be avoided or delayed through proper diabetes control, the mortality due to diabetes-related complications increased by 3% between 2000 and 2019 (4). As the number of adults with diabetes (20–79 years old) is expected to be increased by 45.8 percentage points from 2021 (537 million) to 2045 (783 million) internationally (1), the challenge of reducing the incidence of diabetes and diabetes-related complications through prevention and proper treatment is mounting.

Significant evidence confirms a bidirectional relationship between diabetes and periodontal disease (5). Patients with both diabetes and periodontal disease have a higher likelihood of insulin resistance and a higher hemoglobin A1c level than those without periodontal disease (6, 7), further increasing the risk of diabetes-related complications. Meta-analyzed evidence from randomized clinical trials indicated that periodontal therapy improved diabetes control by reducing the hemoglobin A1c level (8, 9). Recent studies in the United States and Dutch found that patients with diabetes who also had periodontal disease and received periodontal treatment consumed less health expenditure than those without treatment (10, 11). Therefore, identifying periodontal disease among patients with diabetes would be a potential path to reduce the burden of diabetes.

Rich evidence shows that rural areas carry a disproportional burden of poor diabetes control and diabetes-related complications, such as poor hemoglobin A1c, renal failure, and limb amputation across different countries (12, 13). Given a circular relationship between diabetes and periodontal disease, rural patients with diabetes have a higher likelihood of having periodontal disease than those in urban areas. However, existing studies examining rural–urban differences in periodontal diseases primarily focused on the general population or older adults and (14–17) left patients with diabetes unstudied.

There are two aims of the present study. Aim 1 investigates rural–urban disparities in the incidence of periodontal diseases among patients with type 2 diabetes. Aim 2 mainly focused on patients with periodontal disease and compared their differences in treatment intensity of dental care between rural and urban areas. In Aim 1, we hypothesized that among patients with type 2 diabetes, rural patients had a higher incidence of periodontal disease than urban patients, given a circular relationship between diabetes and periodontal disease and a high burden of poor diabetes outcomes in the rural population (5, 12, 13, 18, 19). However, evidence showed that the rural population has fewer dental visits and higher unmet dental needs than the urban population (20–22). If so, periodontal disease is unlikely to be diagnosed among rural patients with diabetes due to the underutilization of dental care. Given this, we proposed an alternative hypothesis— among patients with type 2 diabetes, rural patients have a lower incidence of periodontal disease than urban patients. Evidence showed that underutilization of necessary dental care in rural areas is common (20, 21). Therefore, in Aim 2, we hypothesized that among patients with both diabetes and periodontal disease, rural patients received fewer dental visits than urban patients.

The present study used the national data in Taiwan to test the two hypotheses. Like other countries, Taiwan faces the significant challenge of rising diabetes population. The age-adjusted prevalence of diabetes was 9.7% in 2021 and is expected to reach 12.6% in 2045 (23). More than 99% of the Taiwanese population can access low-cost comprehensive health insurance coverage, including covering inpatient and outpatient care, Chinese medicine, drug, and dental and eye care. However, Taiwan still faces persistent rural–urban disparities in diabetes-related complications (24, 25). About 20% of the Taiwanese population lives in rural areas (26), similar to some developed countries, like Canada, France, and the United States (27). Empirical evidence from the present study fills the existing literature gap and may shed light on narrowing rural–urban disparities in diabetes outcomes and ease the burden of diabetes care.

## Materials and methods

### Study design and data sources

The present study employed a retrospective cohort design to obtain the aims of the present study. Three data sources were merged to test the hypotheses. One is the 2010 Diabetes Mellitus Health Database (DMHD) at the patient level, which contains a cohort of patients who were newly diagnosed with diabetes in 2010. Based on the Department of Statistics, Ministry of Health and Welfare in Taiwan, the 2010 diabetes cohort must meet both criteria: (1) at least three outpatient visits or one hospitalization due to diabetes (the first three International Classification of Disease, Ninth Revision Clinical Modification (ICD-9-CM) codes 250) in 2010, and (2) no previous visits with any diagnoses of diabetes preceding the first visit related to diabetes in 2010 (28). The DMHD contains all claims information of the cohort from year to year, which allows researchers to track healthcare utilization of the cohort. The claims data include hospitalizations, physician visits for Western medicine and Chinese medicine, dental and vision care, and prescriptions. The data also include, but are not limited to, principal and secondary diagnosis codes, procedure codes, prescription codes, and expenses. The present study tracked the 2010 cohort for a decade until 2019.

The other two data sources were at the township level. The National Geographic Information Standardization platform provided the total population and the number of persons with different years of education at the township level. The Department of Statistics, Ministry of Health and Welfare provided the number of dentists for each township.

### Study samples

**Figure 1:**
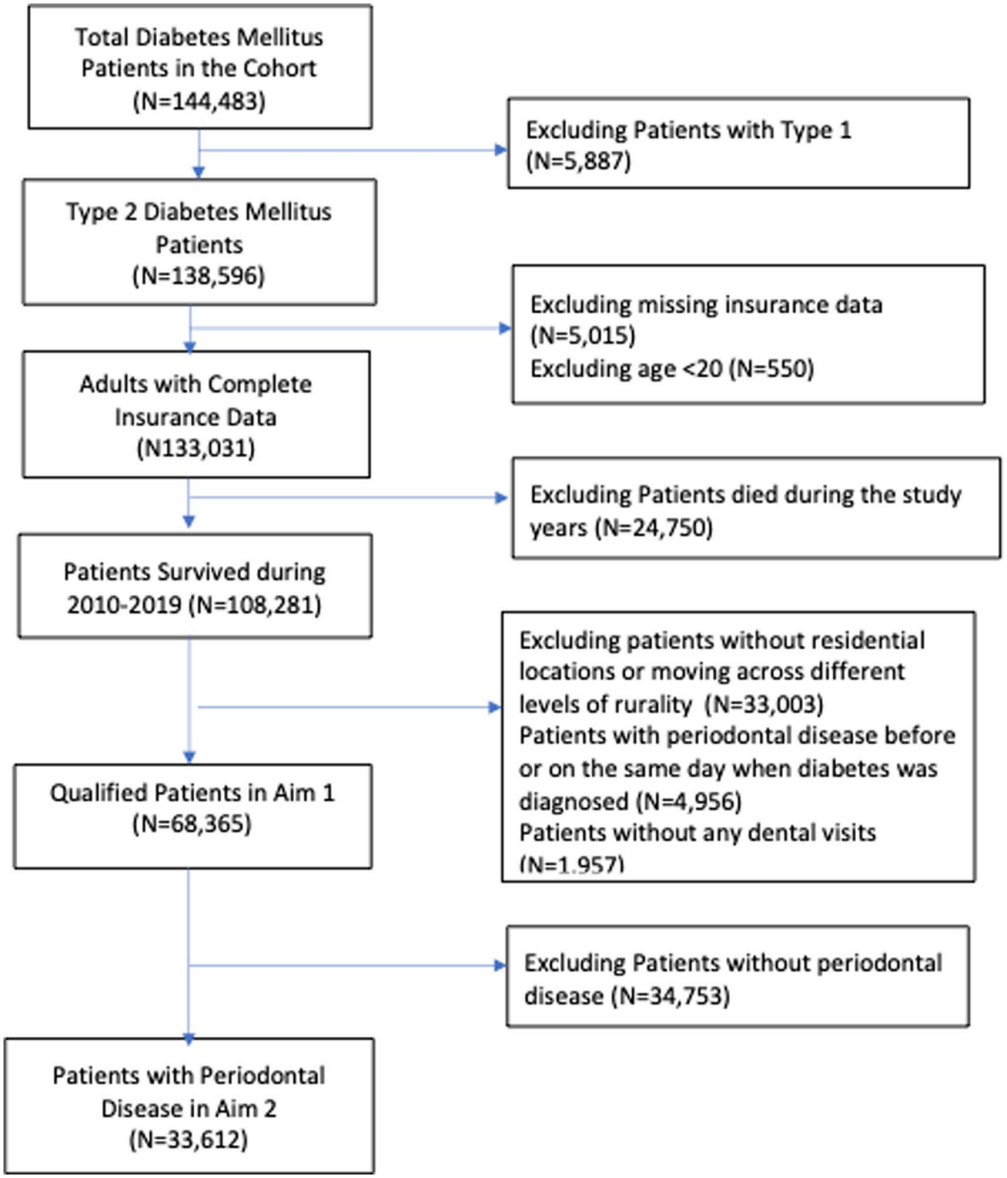
Study sample selection process.

There were two study samples for each aim. In Aim 1, the study sample was extracted from 2010 DMHD and must meet the following criteria: (1) patients with type 2 diabetes and aged 20 and above in 2010; (2) patients without missing data, such as age and residential location in the health insurance registration file; (3) patients who were alive and stayed at the same level of rurality from 2010 through 2019. Finally, we excluded patients without any dental visits during the study period because, without dental visits, we were unable to identify whether or not patients had periodontal disease. In Aim 2, the study sample was extracted from those in Aim 1. We excluded patients with periodontal disease before or on the same day they were diagnosed with diabetes. Figure 1 presents the selection process for the study sample in Aim 1 and Aim 2.

### Variable measures

#### Dependent variables

The primary outcome of interest in Aim 1 was the incidence of periodontal disease. Because the ICD-9-CM was replaced by the ICD-10-CM in 2016, we used ICD-9-CM codes (2012–2015) and ICD-10 CM codes (2016–2019) along with anatomical therapeutic chemical codes to identify periodontal disease. Table 1 lists all the detailed codes used to define periodontal disease. The dependent variable in Aim 2 was the treatment intensity measured by the total number of dental visits during 2010–2019 after patients were diagnosed with periodontal disease.

**Table 1:** ICD-9-CM / ICD-10-CM for periodontal disease

Diagnosed codes		
ICD-9-CM	OR	ICD-10-CM
523.0-523.5 523.6,523.8,523.9		K03.6, K05.00, K05.01, K05.10, K05.11, K05.20, K05.21, K05.22, K05.30, K05.31, K05.32, K05.4, K05.5, K05.6, K06.0, K06.1, K06.2, K06.8, K06.9
And		
Therapeutic codes		
91006C-91008C, 91009B, 91010B, 91011C-91013C, 91103C,91104C, 92033C, P4001C, P4002C, 92013C, 92014C		

### Key independent variable of interest

The key independent variable of interest is the different levels of rurality. Liu and colleagues used demographic characteristics (e.g., population density, percentage of the population with higher education), level of industrialization (i.e., percentage of agriculture workforce), and healthcare resources (i.e., the number of physicians per 100,000 population) to classify all townships in Taiwan into seven clusters for the different degree of urbanization (29). Following previous studies (20, 24, 30), we defined townships at levels 1 and 2 as urban areas, townships at levels 3–5 as suburban areas, and those at levels 6 and 7 as rural areas. We then created two dummy variables for individuals in rural and suburban areas and treated those in urban areas as the reference group.

### Control variables

The Andersen Behavioral Model of Health Care Utilization was applied to define control variables. The model outlined that health outcomes and healthcare utilization are a function of predisposing, enabling, health needs factors at the individual level, and resources at the community level (31). The predisposing factors included age and sex. There were two enabling factors. Income was defined as the base salary used to calculate the premium of NHI in Taiwan. We grouped the base salary into four categories (<USD 667, USD 667-USD 1333, USD >1333, and dependent). We could not know income from the “dependent” category because they relied on one of their family members to join NHI. Education was not available at the individual level. Therefore, the present study used the population without a high school diploma at the township level as a proxy for an individual’s education, which was a continuous variable. The health needs factors included the Charlson Comorbidity Index (CCI), which had been defined in the literature (32, 33). We created two dummy variables to represent patients whose CCI was 1 and whose CCI was two and above, respectively, with those whose CCI was zero as the reference group. Since preventive dental care is negatively associated with the likelihood of periodontal disease, we included the number of preventive dental care visits as one of the covariates when analyzing the incidence of periodontal disease. The community resource at the township level is the number of dentists per 1,000 population.

### Statistical analysis

We utilized Chi-square and ANOVA tests to compare the differences in study variables among rural, suburban, and urban areas without controlling for covariates. In Aim 1, we applied Cox proportional hazards survival model to estimate patients’ hazard ratios (HR) by treating a periodontal disease as a time-to-event outcome, with a right censor at the end of 2019. HR is interpreted as the risk of individuals having the periodontal disease at time t (year in the present study), conditional on those without periodontal disease to time t. In Aim 2, we applied the Poisson regression model to estimate the adjusted rate ratios (RR) of dental visits among patients with both diabetes and periodontal disease across different levels of rurality.

## Results

The total number of patients in the 2010 diabetes cohort was 144,483. After excluding those who did not meet the study criteria, the number of qualified patients remaining in Aim 1 was 68,365 (Figure 1). In Aim 2, the qualified patients in Aim 1 who further developed periodontal disease were 33,612. In other words, about 49% of patients with type 2 diabetes in our study was further diagnosed with periodontal disease within 10 years.

Of 68,365 patients, 1,391 (2.03%), 13,724 (20.07%), and 53,250 (77.89%) persistently stayed in rural, suburban, and urban areas, respectively, during the study period. Among them, 489 (35.15%) in the rural group, 5,529 (40.29%) in the suburban group, and 27,594 (51.82%) in the urban group were further diagnosed with periodontal disease. On average, the number of preventive dental care visits per year was less than one across all three levels of rurality (0.43, 0.62, and 0.89 for rural, suburban, and urban, respectively). In addition, the urban group had a slightly higher percentage of males than the other two groups (52% for the urban group and about 50% for both rural and suburban groups). Additionally, the rural group had a higher percentage of the population aged 65 and above (31.63%) than the suburban (23.90%) and urban groups (20.88%; *p* < 0.001). Furthermore, the rural and suburban groups had a higher percentage of the population without a high school diploma (39% for rural and 34% for suburban) than the urban group (24%; *p* < 0.001). Also, the rural and suburban groups had a lower percentage of income with US $1,333 and above (USD 1 = NTD 30) than the urban group (6.54, 10.99, and 18.28% for rural, suburban, and urban, respectively, *p* < 0.001). Finally, the rural and suburban groups had a lower number of dentists per 1,000 population (3.48 for rural and 4.50 for suburban) than the urban group (7.1 dentists; *p* < 0.001). Table 2 presents the differences in study variables among different levels of rurality.

**Table 2:** Descriptive statistics of the study variables.

Variables	Total	Rural N (%)	Suburban N (%)	Urban N (%)	*p*-value
Key independent variables					
Total	68,365	1,391(2.03)	13,724(20.07)	53,250(77.89)	
Periodontitis					<0.001
No periodontal disease	34,753	902(64.85)	8,195(59.71)	25,656(48.18)	
New periodontal disease	33,612	489(35.15)	5,529(40.29)	27,594(51.82)	
Number of preventive^a^ dental care per year	68,365	0.43(0.84)	0.62 (1.89)	0.89 (1.29)	<0.001
Predisposing variables					
Sex					0.009
Female	32,900	690(49.60)	6,749(49.18)	25,461(47.81)	
Male	3,5,465	701(50.40)	6,975(50.82)	27,789(52.19)	
Age					<0.001
20–44	12,409	188(13.52)	2,339(17.04)	9,882(18.56)	
45–54	20,203	370(26.60)	4,135(30.13)	15,698(29.48)	
55–64	20,916	393(28.25)	3,969(28.92)	16,554(31.09)	
65–74	10,877	301(21.64)	2,383(17.36)	8,193(15.39)	
75+	3,960	139(9.99)	898(6.54)	2,923(5.49)	
Enabling factors					
Percentage of population w/o a HS diploma(mean ± Std)	68,365	0.39 ± 0.06	0.34 ± 0.07	0.24 ± 0.06	<0.001
Insurance premium (USD/month)					<0.001
Less than $667 (Ref.)	13,322	215(15.46)	2,115(15.41)	10,992(20.64)	
Dependent	17,618	199(14.31)	3,226(23.51)	14,193(26.65)	
$667–$1,333	26,091	886(63.70)	6,875(50.09)	18,330(34.42)	
$1,333+	11,334	91(6.54)	1,508(10.99)	9,735(18.28)	
Health need variables					
Charlson Comorbidity Index					0.14
0	66,999	1,355(97.41)	13,439(97.92)	52,205(98.04)	
1	554	19(1.37)	121(0.88)	414(0.78)	
2	812	17(1.22)	164(1.19)	631(1.18)	
Community Resources					
Number of dentists per 1,000 population (mean ± Std)	68,365	3.48 ± 1.25	4.50 ± 1.37	7.06 ± 2.83	<0.001

Table 3 presented the findings from two models. One is the adjusted HR for the incidence of periodontal disease among patients with type 2 diabetes from the Cox proportional hazard regression analysis. The other is adjusted RR for treatment intensity of periodontal disease among patients with type 2 diabetes and further diagnosed with periodontal disease from the Poisson model. After adjusting for the covariates, rural patients (HR = 0.83, 95% CI: 0.75–0.91) and suburban patients (HR = 0.86, 95% CI: 0.83–0.89) had a lower incidence of periodontal disease than urban patients. All covariates were statistically significant, except for sex, aged groups 45–45 and 65–74, and comorbidities. Compared to patients in the aged group of 20–44, those in the aged groups of 55–64 (HR = 1.03, 95%CI: 1.00–1.07) had a higher incidence of periodontal disease while those in the aged group of 75 had a lower incidence (HR = 0.82, 95%CI = 0.78–0.87). Increases in the population without a high school diploma were positively associated with an incidence of periodontal disease (HR = 1.03, 95%CI = 1.02–1.03). Compared to individuals with an income of USD 1,333 and above, those with incomes less than USD 1,333 had a lower risk of periodontal disease (HR ranging from 0.87 to 0.93, with 95% CI ranging from 0.85 to 0.97, *p* < 0.001). Increases in preventive dental care visits are associated positively with an incidence of periodontal disease (HR = 1.29, 95%CI = 1.28–1.29)Finally, increases in the number of dentists per 1,000 population were negatively associated with the incidence of periodontal disease (HR = 0.65, 95%CI: 0.53–0.80, *p* < 0.001).

**Table 3:** Rural–Urban disparities in the incidence and treatment intensity of periodontal diseases from cox and poisson regression models.

	Adjusted HR (95%CI)	Adjusted RR (95%CI)
Key Independent variables		
Rural (ref = Urban)	0.83*** (0.75–0.91)	0.93** (0.88–0.98)
Suburban (ref = Urban)	0.86*** (0.83–0.89)	0.97*** (0.95–0.99)
Predisposing variables		
Sex (ref.: Female)	0.99 (0.91–1.01)	1.05*** (1.03–1.06)
Age (ref.:20–44)		
45–54	1.03 (1.00–1.06)	1.15*** (1.13–1.17)
55–64	1.03* (1.00–1.07)	1.19*** (1.17–1.21)
65–74	0.99 (0.95–1.03)	1.19*** (1.17–1.22)
75+	0.82*** (0.78–0.87)	1.08*** (1.04–1.11)
Enabling variables		
Percentage of population w/o a HS diploma (mean ± Std)	1.03*** (1.02–1.03)	1.00 (1.00–1.00)
Income Status (Ref.: $1,333+)		
Less than $667	0.93*** (0.90–0.97)	0.95*** (0.93–0.96)
Dependent	0.89*** (0.86–0.92)	0.93*** (0.92–0.95)
$667–$1,333	0.87*** (0.85–0.90)	0.92*** (0.90–0.93)
Health need variables		
Charlson Comorbidity Index (ref.: CCI = 0)		
1	1.09 (0.97–1.22)	0.88*** (0.83–0.94)
2+	1.09 (0.99–1.20)	0.99 (0.94–1.04)
Number of preventive dental care	1.29*** (1.28–1.29)	NA
Community resources		
Number of dentists per 1,000 population (mean ± Std)	0.65*** (0.53–0.80)	0.58*** (0.52–0.65)


*p* < 0.05; **p < 0.01; ***p < 0.001.

For the total number of dental visits after patients were diagnosed with periodontal disease, we found that rural and suburban patients were 3–7% fewer dental visits than urban patients after adjusting for the covariates (RR = 0.93, 95%CI: 0.88–0.98 for rural and RR = 0.97, 95% CI: 0.95–0.99 for suburban). Except for education and CCI, all covariates were significant. All aged groups from 45 to 75 and above have 8–19% more dental visits than the aged group 20–44 (RR ranging from 1.08 to 1.19, with 95% CI ranging from 1.04 to 1.22). Furthermore, low-income patients with diabetes and dependents also received 5–8% fewer dental visits than those with income1,333 UDS and above (RR ranging from 0.92 to 0.95, with 95% CI ranging from 0.92 to 0.95). Finally, increases in the number of dentists per 1,000 population decreased the number of dental visits for periodontal disease (RR = 0.58, 95%CI: 0.52–0.65, *p* < 0.001).

## Discussion

Plenty of studies have examined the issues of rural–urban disparities for oral health problems and diabetes individually (12, 18, 20, 34, 35). Few studies investigate rural–urban disparities in oral health-related quality of life for the general population (36, 37). The present study is the first that examined rural–urban disparities in the incidence and treatment intensity of periodontal disease among patients with diabetes.

In summary, the findings in Aims 1 and 2 support the hypotheses based on the underutilization of dental care in rural areas, namely that rural patients with diabetes did not routinely receive dental care; therefore, they were less likely to be diagnosed with periodontal disease than urban patients with diabetes. For those who had periodontal disease, rural patients received fewer dental visits than urban patients. Although we could not exclude the possibility that a low likelihood of having periodontal disease among rural patients with diabetes could have resulted from their good oral health conditions; however, the possibility is low. First, from the supply side, poor downstream social determinants of health, such as the shortage of dental workforce in rural areas, increase the barriers for rural residents to access dental care when necessary (38). In our study, rural areas had only about 50% of the urban dental workforce (3.48 dentists per 1,000 population in rural areas versus 7.06 dentists per 1,000 population in urban areas in Table 2). Second, from the demand side, poor downstream social determinants of health, such as low socioeconomic status and low health literacy among rural residents, prevent rural residents from utilizing necessary dental care (39, 40). In the present study, rural and suburban patients with diabetes had a higher percentage of low-income population and population without a high school diploma than urban patients with diabetes. Most importantly, our findings in Aim 2 also showed that rural patients with diabetes and periodontal disease received fewer dental visits than their counterparts, which supported the assumption that rural patients with diabetes underutilized necessary dental care. Our findings regarding the underutilization of dental care among rural patients with diabetes are consistent with the evidence based on the general population in Taiwan and other countries (20, 21, 41).

Moreover, about 49% of our study sample was diagnosed with periodontal disease within 10 years after they were diagnosed with diabetes. Based on our argument about the underutilization of dental care in rural areas, the likelihood of undiagnosed periodontal diseases among rural patients with diabetes is high. Therefore, the incidence of periodontal disease among patients with diabetes in the present study is likely underestimated. Given a causal relationship that runs both ways between diabetes and periodontal disease (5), undiagnosed periodontal disease and undertreatment of periodontal disease among rural patients with diabetes may partially explain the persistence in rural–urban disparities in poor diabetes control and diabetes-related complications in Taiwan (24, 25, 30). Given the pattern of underutilization of dental care found in rural areas across different countries (21, 41–43), the findings in the present study have a high potential to imply to other countries.

## Limitations

The present study has limitations. First, utilizing claims data to identify periodontal disease prevents the research team from distinguishing the severity of the periodontal disease. Second, we only observed patients with diabetes for 10 years. Patients may develop periodontal disease later, which could not be detected by the present study. Furthermore, we excluded patients with diabetes who had never had a dental visit during our study period since they did not have any claims data for the research team to identify periodontal disease. Compared to patients with dental visits, those without dental visits are more likely to be male and younger and to have income less than USD 667. Therefore, the generalizability of the findings in the present study to the patients without dental visits should be cautious. Future studies investigating the characteristics and other healthcare utilization for patients with diabetes who never have a dental visit are recommended.

Despite the limitations above, the findings in the present provide implications for policies and future research. Underutilizing dental care in rural areas is common even in the system with low-cost or free dental care, like NHI in Taiwan, the Medicaid program for low-income people in the United States, and statutory health insurance in Germany (20, 41, 44). Therefore, health insurance coverage for all at a low cost can only ease rural–urban disparities in dental care to a certain degree. Policy interventions at the system level are needed to facilitate accessing dental care in rural areas. For example, in 2001, Taiwan implemented the diabetes pay-for-performance program that provided financial incentives to providers if their patients received recommended tests or exams (e.g., hemoglobin A1c and eye exams). Consequently, rural–urban disparities in diabetes-related recommended tests were reduced (30). Oral health assessments or referrals are not part of the quality performance metrics of the diabetes pay-for-performance program in Taiwan. Since patients with diabetes need to regularly visit their physician for blood tests and/ or refilling prescriptions, adding an oral health assessment or referral as part of a payment incentive in the diabetes pay-for-performance program would motivate physicians to conduct oral health assessments or referrals for their patients. Additionally, the payment incentive for oral health assessments or referrals at physician’s clinics would foster the medical-dental integrated delivery care recommended by Dale and colleagues and the Department of Health and Human Services Administration (35, 45), which would facilitate moving diabetes care toward patient-centered care.

For future research, we argued a high prevalence of undiagnosed periodontal disease among rural patients with diabetes given the findings in Aim 1. Due to the restriction of claims data, we could not assess the magnitude of differences in undiagnosed periodontal disease between rural and urban patients with diabetes. Evidence showed that patients with diabetes had poor oral health knowledge and behaviors than those without diabetes (46, 47). Given this, the prevalence of undiagnosed periodontal disease is not only high among rural patients with diabetes but also common among urban patients with diabetes. Future studies utilizing patients’ clinical dental data to assess the prevalence of the undiagnosed periodontal disease among patients with diabetes and investigate the difference in the prevalence of undiagnosed periodontal disease between rural and urban patients with diabetes are strongly recommended. Furthermore, NHI offers free care for two preventive dental care visits per year in Taiwan. However, the average of preventive dental care visits was low among patients with diabetes across all levels of rurality. Qualitative studies that explore the reasons why patients with diabetes do not have regular preventive dental care visits are strongly recommended.

## Conclusion

The rising prevalence of diabetes and its complications threatens population health, especially in rural areas. Undiagnosed periodontal disease and undertreatment of periodontal disease among rural patients with diabetes is common, which likely explains the persistence of rural–urban disparities in diabetes. Treating periodontal disease for patients with diabetes is life- and cost-saving and is a potential path to reduce the threat of diabetes on population health. Therefore, identifying periodontal disease for treatment at the early stage for patients with diabetes is crucial. Policy interventions that motivate providers to form the medical-dental delivery model would increase dental care utilization for all patients with diabetes and move diabetes care toward patient-centered care, which would eventually ease the threat of diabetes on population health.

## Data availability statement

The data analyzed in this study is subject to the following licenses/restrictions: the datasets used and analyzed in the present study are not publicly available but are available from the Taiwan National Health Insurance Program in Taiwan. Restrictions apply to the availability of these data, which were used under the license for the present study. Data are available from https://nhird.nhri.org.tw with the permission of the Taiwan National Health Insurance (NHI) program. Requests to access these datasets should be directed to chenhf@kmu.edu.tw.

## Ethics statement

The studies involving humans were approved by Institutional Review Board at Kaohsiung Medical University Hospital in Taiwan (KMUHIRB- E(I)-20210211). The studies were conducted in accordance with the local legislation and institutional requirements. Written informed consent for participation was not required from the participants or the participants’ legal guardians/next of kin in accordance with the national legislation and institutional requirements.

## Author contributions

HFC conceptualized the study, obtained funding, designed the study, conducted a quality check, wrote the first draft of the manuscript, and revised the manuscript. HEL conceptualized the study, designed the study, interpreted the findings, supervised the project, and reviewed and revised the manuscript. ITC conceptualized the study, interpreted the findings, reviewed and revised the manuscript, and administered the project. YTH conceptualized the study, managed and analyzed the data, interpret the findings, and reviewed and revised the manuscript. PSH conceptualized the study, interpret the findings, and reviewed and revised the manuscript. SAK conceptualized the study, reviewed literature, interpret the findings, and reviewed and revised the manuscript. All authors contributed to the article and approved the submitted version.

## Funding

The Taiwan National Science and Technology Council (NSTC, previous name is Ministry of Science and Technology, MOST110-2410-H-037-018-MY3) and the Yuan’s General Hospital (YUAN-IACR-22-04) in Taiwan supported this study. We are grateful to the NSTC and the Yuan’s General Hospital for providing funding support.

## Conflict of interest

The authors declare that the research was conducted in the absence of any commercial or financial relationships that could be construed as a potential conflict of interest.

## Publisher’s note

All claims expressed in this article are solely those of the authors and do not necessarily represent those of their affiliated organizations, or those of the publisher, the editors and the reviewers. Any product that may be evaluated in this article, or claim that may be made by its manufacturer, is not guaranteed or endorsed by the publisher.
